# Neural Correlates of Knee Extension and Flexion Force Control: A Kinetically-Instrumented Neuroimaging Study

**DOI:** 10.3389/fnhum.2020.622637

**Published:** 2021-02-04

**Authors:** Dustin R. Grooms, Cody R. Criss, Janet E. Simon, Adam L. Haggerty, Timothy R. Wohl

**Affiliations:** ^1^Ohio Musculoskeletal and Neurological Institute, Ohio University, Grover Center, Athens, OH, United States; ^2^Division of Athletic Training, School of Applied Health Sciences and Wellness, College of Health Sciences and Professions, Ohio University, Grover Center, Athens, OH, United States; ^3^Division of Physical Therapy, School of Rehabilitation and Communication Sciences, College of Health Sciences and Professions, Ohio University, Grover Center, Athens, OH, United States; ^4^Translational Biomedical Sciences Program, Heritage College of Osteopathic Medicine, Ohio University, Athens, OH, United States; ^5^Honors Tutorial College, Ohio University, Athens, OH, United States; ^6^Division of Physical Therapy, School of Health and Rehabilitation Sciences, Ohio State University, Columbus, OH, United States

**Keywords:** force sense, functional magnetic resonance imaging, quadriceps, hamstring, lower extremity, sensorimotor control

## Abstract

**Background**: The regulation of muscle force is a vital aspect of sensorimotor control, requiring intricate neural processes. While neural activity associated with upper extremity force control has been documented, extrapolation to lower extremity force control is limited. Knowledge of how the brain regulates force control for knee extension and flexion may provide insights as to how pathology or intervention impacts central control of movement.

**Objectives**: To develop and implement a neuroimaging-compatible force control paradigm for knee extension and flexion.

**Methods**: A magnetic resonance imaging (MRI) safe load cell was used in a customized apparatus to quantify force (N) during neuroimaging (Philips Achieva 3T). Visual biofeedback and a target sinusoidal wave that fluctuated between 0 and 5 N was provided *via* an MRI-safe virtual reality display. Fifteen right leg dominant female participants (age = 20.3 ± 1.2 years, height = 1.6 ± 0.10 m, weight = 64.8 ± 6.4 kg) completed a knee extension and flexion force matching paradigm during neuroimaging. The force-matching error was calculated based on the difference between the visual target and actual performance. Brain activation patterns were calculated and associated with force-matching error and the difference between quadriceps and hamstring force-matching tasks were evaluated with a mixed-effects model (*z* > 3.1, *p* < 0.05, cluster corrected).

**Results**: Knee extension and flexion force-matching tasks increased BOLD signal among cerebellar, sensorimotor, and visual-processing regions. Increased knee extension force-matching error was associated with greater right frontal cortex and left parietal cortex activity and reduced left lingual gyrus activity. Increased knee flexion force-matching error was associated with reduced left frontal and right parietal region activity. Knee flexion force control increased bilateral premotor, secondary somatosensory, and right anterior temporal activity relative to knee extension. The force-matching error was not statistically different between tasks.

**Conclusion**: Lower extremity force control results in unique activation strategies depending on if engaging knee extension or flexion, with knee flexion requiring increased neural activity (BOLD signal) for the same level of force and no difference in relative error. These fMRI compatible force control paradigms allow precise behavioral quantification of motor performance concurrent with brain activity for lower extremity sensorimotor function and may serve as a method for future research to investigate how pathologies affect lower extremity neuromuscular function.

## Introduction

Determining how the central nervous system regulates force is vital for understanding the neural control of biomechanical action. The integration of neuroimaging techniques with simultaneous biomechanical recording has allowed for concurrent capture of joint position and force with neural activity (Liu et al., 2000; Ward et al., 2008; Naufel et al., 2019). However, the majority of investigations have focused on the upper extremity and the primary motor cortex to elucidate the relationship between muscle force and neural activity (Georgopoulos et al., 1992; Ashe, 1997; Ward et al., 2008). Studies examining neural activity associated with lower extremity motor control have not quantified motor performance beyond movement timing (Luft et al., 2002; Kapreli et al., 2007; Grooms et al., 2019) or have been limited to electroencephalography paradigms, which provide excellent temporal resolution but lack the spatial resolution of functional magnetic resonance imaging (fMRI; Poortvliet et al., 2015). Prior work specific to fMRI has examined neural correlates of quadriceps force regulation in patients with knee osteoarthritis (Shanahan et al., 2015) using an isometric, force-matching paradigm, finding an anterior shift of the knee representation within the primary motor cortex in those with knee osteoarthritis. Various research groups have also employed cycle ergometers (Mehta et al., 2009), gait simulations (Jaeger et al., 2016), or leg press (Grooms et al., 2019) movement paradigms to quantify lower extremity movement with brain imaging. While these paradigms demonstrated success to activate the sensorimotor network and do so reliably, many fMRI lower extremity paradigms are metronome-paced and do not attempt to quantify motor performance (Luft et al., 2002; Kapreli et al., 2006). Therefore, the development of lower extremity paradigms that can concurrently measure neural activity *via* fMRI and biomechanical performance may offer more precise methods to investigate central strategies for force regulation, with implications for pathologies affecting sensorimotor control of the lower extremity (Hortobágyi et al., 2004; Ward et al., 2019).

Prior biomechanically isolated work has demonstrated force control deficits in a variety of orthopedic and neurological pathologies of the lower extremity (Hortobágyi et al., 2004; Docherty and Arnold, 2008; Telianidis et al., 2014) but a clear brain-behavioral interaction has yet to be established (Baumeister et al., 2011). Further, no study to our knowledge has attempted to contrast how the brain regulates force when engaged in knee extension (quadriceps-dominant activity) relative to knee flexion (hamstring-dominant activity). Unique deficits in quadriceps and hamstring function have been reported in a variety of orthopedic and neurological conditions, and the restoration of respective muscle and joint function is vital for the recovery and resumption of activities of daily living, adequate mobility, and mitigating the development of chronic conditions such as osteoarthritis (Manini et al., 2007; Manini and Clark, 2012; Tourville et al., 2014; Arhos et al., 2020). As lower extremity pathologies have been found to manipulate both quadriceps and hamstring muscle activity, timing, and function, determining the neural mechanisms for each is vital to better understand how lower extremity motor control is centrally governed (Telianidis et al., 2014; Abourezk et al., 2017; Blackburn et al., 2017; Hohmann et al., 2019). Isolating neural correlates of quadriceps and hamstring force generation and control may highlight central mechanisms for function following injury and permit the development of novel therapies that restore function. Therefore, our purpose was to: (1) develop and test a lower extremity neuroimaging paradigm for knee extension and flexion force control to better understand how the nervous system regulates lower extremity forces; and (2) determine differences between knee extension and flexion neural activity during a force control task.

## Materials and Methods

### Participants

This study was approved by Ohio University’s Institutional Review Board and all participants signed the informed consent document. We included female recreational athletes (at least 3 h of moderate to vigorous exercise per week, including 1 h of running, cutting, pivoting, or decelerating every week) aged 18–30 years. This population was selected for the following investigative work as they are at unique increased risk for noncontact knee injuries, whereby during athletics, exercise, or activities of daily living that require rapid movement, sensorimotor control of the knee is compromised, resulting in positions that put excessive strain on the joint ligaments (Beynnon et al., 2014; Montalvo et al., 2019).

A sample size estimate was calculated based on effects reported by Shanahan et al. (2015) for the correlation to force-error, and Trinastic et al. (2010) for the contrast between movement conditions. For the force-error correlate analysis, an *r* = 0.83 was reported for the relationship between error and motor cortex peak activation location (Shanahan et al., 2015). A sample size estimate was calculated based on *r* = 0.83, α = 0.05, and 1 − β = 0.8 indicating a total sample size of 8 is required. For the motor condition analysis, the effect size between ankle plantarflexion and dorsiflexion was calculated as *d* = 1.42 (Trinastic et al., 2010). A sample size estimate was calculated based on *d* = 1.42, α = 0.05, and 1 − β = 0.8 resulting in needing a sample size of 7. Additionally, we modeled our study on previous literature of Newton et al. (2008) and Mehta et al. (2009) regarding paradigm development who enrolled 9 and 10 participants, respectively. Therefore, enrolling 15 participants provided adequate power for the proposed study. We enrolled 15 participants (15 F; age = 20.3 ± 1.2 years, height = 1.6 ± 0.10 m, and weight = 64.8 ± 6.4 kg) in this study. All participants were right leg dominant and met the exercise requirement criteria, as determined by the Marx Activity Rating Scale (Table 1; Marx et al., 2001).

**Table 1:** Demographics and force error.

Data	Mean ± SD
Age (years)	20.3 ± 1.2
Height (m)	1.6 ± 0.10
Weight (kg)	64.8 ± 6.4
Activity level (Marx)	9.93 ± 5.50
Run	3.00 ± 0.85
Cut	2.07 ± 1.62
Decelerate	2.40 ± 1.64
Pivot	2.27 ± 1.49
Knee extension error (*N*)	1.068 ± 0.327
Knee flexion error (*N*)	0.999 ± 0.189

We excluded participants who were contraindicated for fMRI (e.g., pregnancy, implanted metal devices, claustrophobia, and any other criteria as determined by the MRI operator), have a visual impairment, have a history of seizures or epilepsy, or have a history of surgery on the back, hip, leg, knee, etc. Other screening criteria included: primary sport, leg dominance, previous leg injury, medical history anxiety disorder, ADHD, depression, diabetic neuropathy, concussion or traumatic brain injury, cerebral palsy, balance disorder, vertigo, Parkinson’s disease, multiple sclerosis, substance abuse or dependence, heart disease/defect, and prescription medication use within the 24 h before data collection. No individuals reported any of the previous medical conditions or consumed any medications impacting the data collection.

### fMRI Data Collection

Data collection was completed in a single neuroimaging session (~45 min including set-up, instruction, and scan time). During imaging, all participants wore standardized shorts and socks without shoes to reduce the possibility of altered skin tactile feedback. Participants also wore a splint to lock their right (dominant leg) ankle at neutral (~90°) to minimize ankle movement throughout the scan. Headphone and hearing protection was provided for subject comfort and safety and to facilitate communication during scanning. While lying supine in the fMRI scanner, participants were strapped down to the table with four straps, one across the thighs at the mid-point between the greater trochanter and knee joint line, one across the hips at the anterior superior iliac spines, and two across the chest, from each shoulder to the pelvis at the iliac crest. The knee was fixed near terminal extension between 10° and 15° of flexion. Participants were also fitted with customized padding to reduce head motion. This padding was high-density MRI-safe foam that was inserted around the sides and top of the head to remove space between the skull and head coil. This was customized based on skull size, with those with larger skulls requiring less padding and smaller skulls requiring more padding.

fMRI scans were collected with a 16-channel head coil. Before the functional data collection, a three-dimensional high-resolution T1-weighted image (repetition time (TR): 2,000 ms, echo time (TE): 4.58 ms, field of view: 256 × 256 mm; matrix: 256 × 256; slice thickness 1 mm, 176 slices, 8° flip-angle) was collected for image registration (~8 min). fMRI collection parameters include 10 whole-brain gradient-echo-echo planar scans per block (four force-matching blocks, five rest blocks) acquired with a 3 s TR with anterior-posterior phase encoding and a 3.75 × 3.75 in-plane resolution, 5 mm slice thickness for 38 axial slices with a 35 ms TE, 90° flip angle, the field of view 240 mm and 64 × 64 matrix. Each functional force-matching run lasted 4 min and 30 s. fMRI measured regional brain activity during rest and motor control conditions, which were contrasted to isolate the regional brain activity to the isometric knee extension and flexion force-matching tasks.

**Figure 1:**
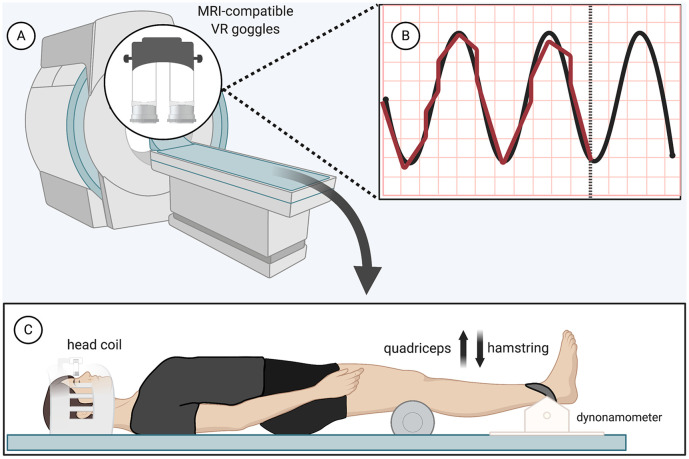
Functional magnetic resonance imaging (fMRI) force-matching task—**(A)** magnetic resonance imaging (MRI)-compatible virtual reality display **(B)** black, Sinewave graph (0–5 N, 1.2 Hz); red, real-time display of participant force **(C)** load cell apparatus and patient position (participant restraints not pictured). Created with Biorender.

The isometric force-matching motor task required the participant to either “kick up” or “press down” against a load cell (Biopac Systems Inc., TSD121B-MRI, 1,000 Hz sampling frequency) at the ankle (Figure 1C). Both knees rested upon a foam roller, while only the dominant, right leg was additionally strapped to a device against the load cell. Participants had to match their force output (visualized with biofeedback provided by MRI-safe virtual reality) with a sine wave that oscillated (1.2 Hz) from 0 to 5 N for 30 s with 30 s of rest for four total cycles, resulting in four force-matching blocks interspersed with five rest blocks of 30 s each (with the paradigm starting and ending with rest) for a total run time of 4 min and 30 s (Figures 1A,B). Standardized auditory cues informed participants when to begin and end force-matching. The force-matching error was calculated based on the difference between the visual target (sine wave) and actual performance (biofeedback). The force level for this study was low and we recruited a young active cohort to minimize the potential influence of fatigue; however, fatigue was monitored regularly and breaks were offered. No participants indicated fatigue or needed a break beyond the few minutes between scans.

Participants practiced the force-matching task for a full run with immediate examiner feedback if instructions were not understood before completing the task during scanning. Also before data collection at the MRI, participants completed a mock MRI session where they familiarized themselves with the MRI environment, restraints to reduce head motion, and the lower extremity motor task. The participants were permitted to ask questions and practice the tasks with feedback from the experimenter. The practice session included three practice blocks (30 s each) of each force-matching task with examiner cueing to ensure the participant understood the task, followed by a complete run of each task with the same feedback and timing as during the actual MRI data collection session.

### Error Calculation and Statistical Analysis

The force-matching error was recorded continuously throughout the force-matching tasks. For statistical analysis, error across the 30-s blocks was partitioned into 3-s intervals (the time interval for one sine wave). The first 3-s interval of each 30-s block was removed from the data analysis, as participants commonly required a few seconds to become acclimated to the task during the initiation of the movement block and thus, was shown to bias the overall average of the remaining nine intervals. The average error for each block was determined by the root mean square of the differential from target force to actual force on the remaining nine sets of 3-s intervals within each block, and the average error across the four blocks was computed for each participant for statistical analyses. Average knee extension and flexion force-matching error were compared with a paired samples *t*-test with an alpha set at 0.05.

### fMRI Data and Statistical Analysis

The fMRI technique used in this study quantified the blood-oxygen-level-dependent (BOLD) signal *via* the hemodynamic response by contrasting the respective force-matching condition with interspersed rest conditions (Friston et al., 1995). We controlled for the additional sensory feedback of the strap across the shank by ensuring it was tightly pressed during both the rest and force-matching conditions, but the pressure of this tactile stimulus unavoidably changes with contraction and may thus contribute to the overall BOLD response. The BOLD response, quantified *via* fMRI collection and analysis, has been validated against direct neural recordings, demonstrating a very high correlation between blood flow and neural activity (Logothetis et al., 2001; Goense and Logothetis, 2008). The reliability of fMRI quantification of the BOLD signal is generally high and specific to knee movement and has high inter-session reliability (Newton et al., 2008; McGregor et al., 2012).

The fMRI statistical analyses were performed using the Oxford Centre for Functional MRI of the Brain Software Library (Smith et al., 2004; Jenkinson et al., 2012). Image analysis began with standard pre-statistic processing applied to individual data in the standardized FSL recommended order (Jenkinson et al., 2012), which included nonbrain removal, slice timing correction, standard motion correction, and realignment parameters (three rotations and three translations) as covariates to limit confounding effects of head movement and spatial smoothing at 6 mm before statistical analysis (Jenkinson et al., 2002). One participant was removed from the knee extension force-matching analysis due to excessive head motion (>0.5 mm) and two removed from the knee flexion force-matching analysis, resulting in *n* = 14 for knee extension, *n* = 13 for knee flexion, and *n* = 13 for comparison between knee extension and knee flexion. High-pass temporal filtering at 90 Hz and time-series statistical analyses were carried out using a linear model with local autocorrelation correction. Functional images were co-registered with the respective high-resolution T1 image and the standard Montreal Neurological Institute template 152 using linear image registration. This registration process allowed data from each participant to be spatially aligned on a standardized brain template for comparison.

The subject-level analysis of knee sensorimotor control relative to rest was completed using a *z* score greater than 3.1 and a (corrected) cluster significance threshold of α < 0.05. The cluster correction for multiple comparisons uses a variant of the Gaussian random field theory to decrease type I error in the statistical parametric mapping of imaging data by evaluating the activation not only at each voxel but also at the surrounding voxel cluster (as it is unlikely that the voxel tested and surrounding voxels are active above the threshold due to chance; Poldrack et al., 2011). The paired contrast between each individual’s quadriceps vs. hamstring force control neural activity was performed with group *z* statistic images set at a threshold of *z* scores of greater than 3.1 and a corrected cluster significance level of α < 0.05. As this was a brain activity correlate identification study, the effect size (*r*-value) of the relationship between brain activity and behavior are not reported to avoid circularity (voxel selection and magnitude estimation on the same data) and a follow-up validation study is required to estimate effect size with the identified regions from this work (Kriegeskorte et al., 2009, 2010).

## Results

Regional brain activation is reported as contralateral [indicating activation on the opposite side of the task, or the left hemisphere, as the task was always completed with the right (dominant) lower extremity] or ipsilateral (being the same side as the task, or the right hemisphere; Tables s 2–4). Regions of brain activity are reported that were identified in FSLeyes based on peak-voxel with the Harvard-Oxford Cortical and Subcortical Structural Atlas (Desikan et al., 2006), Juelich Histological Atlas (Eickhoff et al., 2006, 2007) and the Cerebellar Atlas in MNI152 space after normalization with FNIRT (Diedrichsen et al., 2009) and with FSL tool atlasquery (Jenkinson et al., 2012). The atlasquery function from FSL utilizes the averaged probability across all voxels in the cluster to identify probabilistic anatomy across the cluster ensuring reporting of peak voxel location and overall cluster spatial representation.

**Table 2:** Regions of increased brain activity during the knee extension force-matching task.

Cluster index	Brain regions	Voxel count	*P*-value	Peak MNI voxel			*Z* stat-max
				*x*	*y*	*z*	
Overall activation during knee extension force-matching							
6	B Precentral gyrus, Postcentral gyrus, Superior parietal lobule,	17,662	<0.00001	0	−34	56	10.1
	Lateral occipital cortex
5	B Precentral gyrus, Corticospinal tract, R Thalamus	1,567	<0.00001	10	−16	4	4.99
4	Corticospinal tract, L Thalamus	1,137	<0.00001	−8	−18	16	5.57
3	Precentral Gyrus, Inferior frontal gyrus, Premotor cortex	375	0.000116	−56	0	38	7.22
2	R Cerebellum VIIIA, VIIB, IX	284	0.000882	32	−50	−48	4.79
1	L Cerebellum VIIB, VIIIA, VIIIB, IX	182	0.0113	−20	−70	−44	6.26
Neural activity increase associated with knee extension force error							
3	L Postcentral gyrus, Superior parietal lobule	206	0.00598	−20	−40	76	4.89	<
2	R Frontal pole	142	0.0344	30	52	20	5.02
1	R Middle frontal gyrus	130	0.0489	46	12	40	4.24
Neural activity decrease associated with knee extension force error							
1	Intracalcarine cortex, Lingual gyrus	161	0.0201	−14	−82	10	4.68

*Regions of brain activity are reported that were identified in FSLeyes with the Harvard-Oxford cortical and subcortical structural atlas, Julich histological atlas, and the Cerebellar atlas in MNI152 space after normalization with FNIRT by peak voxel and with FSL tool atlasquery. B, bilateral; L, left; R, right*.

**Table 3:** Regions of increased brain activity during the knee flexion force-matching task.

Cluster index	Brain regions	Voxel count	*P*-value	Peak MNI voxel			*Z* stat-max
				*x*	*y*	*z*	
Overall activation during knee flexion force-matching							
3	B Postcentral gyrus, Precentral gyrus, Superior parietal lobule, Lateral occipital cortex, Supplementary motor cortex, Cingulate gyrus	16,647	<0.00001	−42	−78	−8	11.3
2	B Precentral gyrus, Supramarginal gyrus, Lateral occipital cortex, Lingual gyrus, Occipital fusiform gyrus, Cerebellum Right I-V, VIIB, VIIIA, Left VIIB, VIIIA	12,626	<0.00001	24	−70	−56	10.3
1	R Frontal pole, Frontal orbital cortex	189	0.008	26	34	−22	4.91
Neural activity decrease associated with knee flexion force error							
2	R Precuneus, Postcentral gyrus, Posterior cingulate gyrus, Superior parietal lobule	257	0.00138	6	−40	50	5.91
1	L Frontal pole, Superior frontal gyrus, Middle frontal gyrus	215	0.00402	−36	30	40	5.25

*Regions of brain activity are reported that were identified in FSLeyes with the Harvard-Oxford cortical and subcortical structural atlas, Julich histological atlas, and the cerebellar atlas in MNI152 space after normalization with FNIRT by peak voxel and with FSL tool atlasquery. There was no significant increased neural activity associated with knee flexion error. B, bilateral; L, left; R, right*.

**Table 4:** Regions of difference between knee extension and flexion force-matching.

Cluster index	Brain regions	Voxel count	*P*-value	Peak MNI voxel			*Z* stat-max
				*x*	*y*	*z*	
Increased neural activity knee flexion > knee extension force control							
6	L Precentral and Postcentral gyrus	741	<0.00001	−62	−8	42	5.99
5	L Middle temporal gyrus, Angular gyrus, Inferior parietal lobule	259	0.00112	−62	−52	16	5.19
4	R Temporal pole	245	0.0016	44	22	−34	5.55
3	R Superior temporal gyrus, Supramarginal gyrus, Middle temporal gyrus	239	0.00187	48	−38	2	5.16
2	L Supplementary motor cortex, Paracingulate gyrus	194	0.00619	−6	10	48	4.44
1	B Corticospinal tract, L Thalamus	134	0.0354	−4	−10	−6	4.17

*Regions of brain activity are reported that were identified in FSLeyes with the Harvard-Oxford cortical and subcortical structural atlas, Julich histological atlas, and the cerebellar atlas in MNI152 space after normalization with FNIRT by peak voxel and with FSL tool atlasquery. B, bilateral; L, left; R, right*.

**Figure 2:**
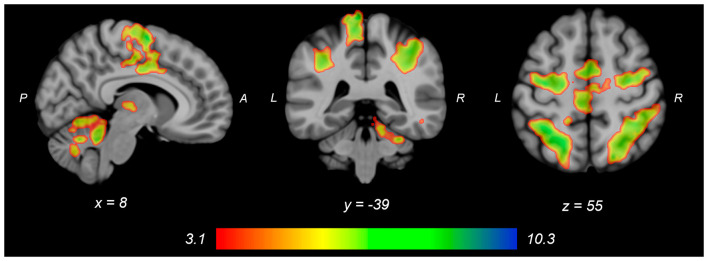
Group average neural activity for knee extension force-matching from Table 2. P, posterior; A, anterior; L, left; R, right.

**Figure 3:**
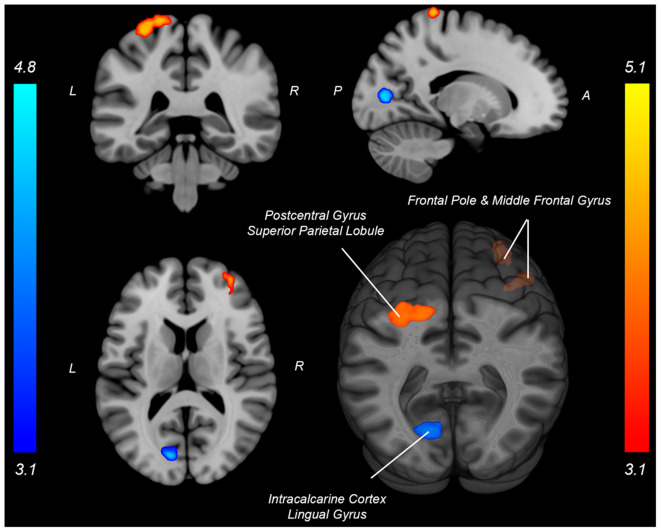
Neural activity associated with knee extension force-matching error from Table 2 (Red: brain activity positively associated with an error. Blue: brain activity negatively associated with an error). P, posterior; A, anterior; L, left; R, right.

**Figure 4:**
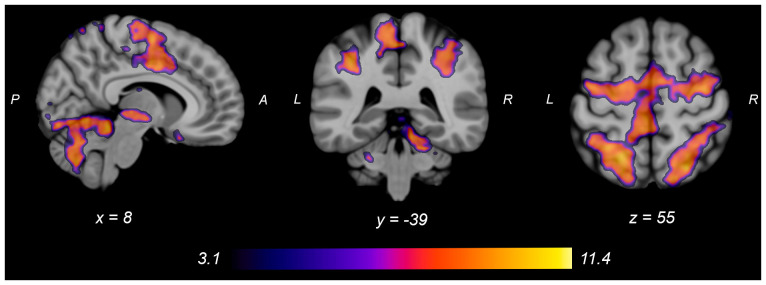
Group average neural activity for knee flexion force-matching from Table 3. P, posterior; A, anterior; L, left; R, right.

**Figure 5:**
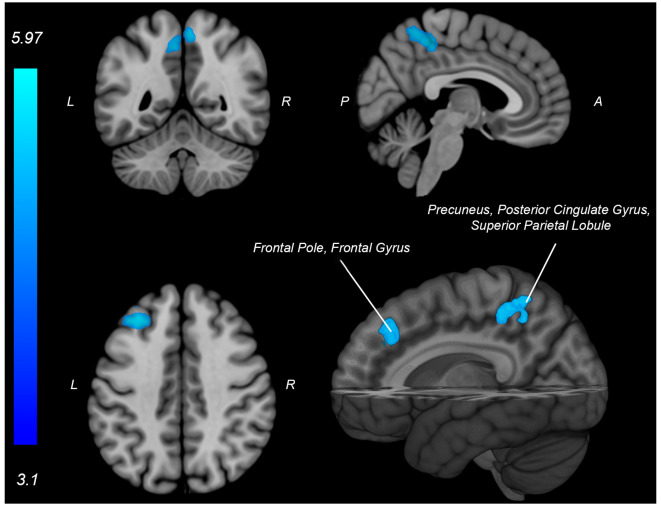
Neural activity (blue) is negatively associated with knee flexion force-matching error from Table 3. P, posterior; A, anterior L, left; R, right.

**Figure 6:**
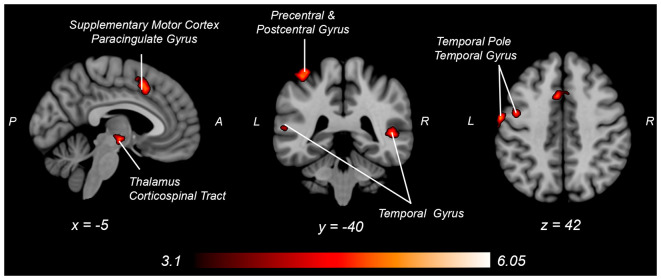
Neural activity increases with knee flexion force matching relative to knee extension from Table 4. P, posterior; A, anterior L, left; R, right.

Both knee extension and flexion force-matching tasks elicited increased bilateral BOLD signal among cerebellar, sensorimotor, and visual-processing regions (Figures 2, Figure 4). Increased knee extension force-matching error was associated with increased BOLD signal within the ipsilateral frontal cortex and contralateral parietal cortex and decreased contralateral BOLD signal within the lingual gyrus and intracalcarine cortex (Figure 3). Increased knee flexion force-matching error was related to decreased contralateral frontal and ipsilateral parietal region activity (Figure 5). Knee flexion force control had increased bilateral premotor, secondary somatosensory, and right anterior temporal activity relative to knee extension force control (Figure 6). Force-matching error performance was not statistically different between the knee extension and flexion tasks (Table 1). Head motion during the knee extension task was: 0.28 ± 0.17 mm absolute motion and 0.11 ± 0.10 mm relative motion. Head motion during the knee flexion task was: 0.22 ± 0.13 mm absolute motion and 0.10 ± 0.09 mm relative motion.

## Discussion

Lower extremity force control results in unique neural activation strategies depending on if engaging the quadriceps for knee extension or the hamstrings for knee flexion, with knee flexion requiring more sensorimotor neural activity for the same level of force generation and relative error. This paradigm allows precise behavioral quantification of motor performance concurrent with brain activity for lower extremity sensorimotor function, which may serve as a method for future research to investigate how pathologies or interventions affect lower extremity neuromuscular function.

### Neural Correlates of Knee Extension Force Control

Knee extension force-matching had a neural activation pattern similar to prior reports of lower extremity knee-focused and quadriceps-dominant movements, with activation across the cortical and subcortical sensorimotor network (Luft et al., 2002; Kapreli et al., 2007). Quadriceps force error was associated with increased activity in frontal and parietal regions and associated with decreased crossmodal (Calvert, 2001) region activity (intracalcarine cortex and lingual gyrus) along the border of the occipital and parietal cortex.

Increased activation of frontal regions with increased error could indicate force control is more complex for those with a higher force-matching error, as previous research has identified an association between increased frontal activity with increased task complexity error (Schubotz and von Cramon, 2002; Mehta et al., 2012; Dunst et al., 2014). It is also possible that as a participant began to perform poorly and visualize their error, they engaged in more extensive or rapid recalibration to attempt to remain on target, requiring greater levels of attentive neural processing (Tracy, 2007; Tracy et al., 2007; Baweja et al., 2009). However, despite increased neural activity among attention and executive function-related brain regions, the relative error was higher which could also be simply a byproduct of more actively attending to their mismatched biofeedback and not secondary to employing a strategy to correct it (Tracy, 2007).

By contrast, those with less force-matching error had increased crossmodal visual-spatial and somatosensory region processing (or increased error had decreased relative activity), which may be involved in aligning and maintaining visual feedback with force regulation from peripheral afferent signals to minimize discrepancy. Previous work within the upper extremity has identified the lingual gyrus and intracalcarine regions to respond to congruent visual and somatosensory feedback (crossmodal; Driver and Spence, 1998; Macaluso et al., 2000). Further, extrastriate activity in the lingual gyrus and intracalcarine cortex has been implicated to be involved in body perception, and active during both visual and limb movements (Astafiev et al., 2004). Therefore, increased extrastriatal activity may correspond with a superior ability to align visual stimuli with proprioceptive afferent signals to minimize force-matching discrepancy. However, the increased extrastriatal activity could also be secondary to visualizing good performance *via* alignment of the target and participant force and not be the mechanism for reduced error. As intracalcarine cortex and lingual gyrus have greater levels of activity when such crossmodal stimuli are congruent compared to incongruent stimuli (e.g., spatial and temporal correspondence of visual presentation and tactile stimulation) and low error results in a visual stimulus that is congruent with proprioceptive sensed force generation and tactile cues (Driver and Spence, 1998; Macaluso et al., 2000).

### Neural Correlates of Knee Flexion Force Control

The knee flexion force-matching task also had a neural activation pattern similar to prior lower extremity neuroimaging paradigms, with activation across the cortical and cerebellar sensorimotor network (Jaeger et al., 2014; Grooms et al., 2019). Knee flexion force error was associated with decreased activity in frontal and parietal regions, however, no increased neural activity was associated with knee flexion error. This contrasts with the knee extension force control error, which had increased frontal and parietal lobe activity associated with increased force error.

This opposition may seem contradictory as one might expect more general alignment for the neural activity underlying force error between knee flexion and extension activities. However, the musculature enabling isolated knee flexion (primarily hamstring) vs. isolated knee extension (primarily quadriceps) have unique neural representation, peripheral nerve innervation, and spinal reflex structure (Jennings and Seedhom, 1994; Mrachacz-Kersting et al., 2006). Thus, the brain differences for error correction between knee extension and flexion may be secondary to mediation at the spinal level. The hamstrings are also typically weaker than the quadriceps (Wyatt and Edwards, 1981; Aagaard et al., 1995; Pincivero et al., 1997) and have a greater proprioceptive error (Relph and Herrington, 2016), potentially secondary to decreased relative cortical representation (Davies, 2020) and less muscle spindle innervation relative to the quadriceps (Banks, 2006).

Anecdotally, the participants in this study had a more difficult time learning how to perform the knee flexion task relative to the knee extension task as many needed more practice trials for the hamstring task than the quadriceps task to achieve reliable performance. The constrained action hypothesis posits that when you attend to a motor task, you constrain the automatic, implicit motor programs that would have otherwise facilitated the movement (Wulf et al., 2001; Kal et al., 2013; Vidal et al., 2018). However, if there is no automatic, implicit motor plan present to guide the movement, then attention to the motor task may improve performance. Therefore, the knee extension task may have been more “intuitive” (implicit) in this sample, contributing to decreased frontal cortex activity not constraining the automatic motor program and facilitating reduced extension error. Conversely, if the knee flexion task is anecdotally less implicit (lacking a well-established, implicit motor program), the association between increased frontal activity and improved performance for flexion may be attributable to the necessity of cognitive-attentive neural processes to drive the motor plan.

### Neural Activity Differences Between Knee Extension and Flexion Force Control

Engaging in knee flexion force control required increased cortical and subcortical activation, including primary sensorimotor cortex, secondary motor cortex, temporal regions, parietal supramarginal gyrus, and corticospinal tract, whereas no brain regions had increased activity for relative knee extension force control. These findings may partially explain the apparent paradoxical similar activation pattern associated with increased knee extension force error, yet decreased knee flexion force error, as the knee flexion force-matching task required greater overall neural activity for similar force-matching performance. This could be secondary to the relatively greater demand on the hamstrings, as they are typically weaker than the quadriceps, requiring elevated neural activity to produce the same force level. Alternatively, the position of the knee may have influenced the result as a near-terminal extension may bias toward quadriceps shortened position and improved steadiness (Krishnan et al., 2011) compared to the hamstring position (lengthened). A likely neurophysiologic contributor is the relatively increased spinal reflexive innervation of the hamstring (Shahani and Young, 1971; Roy et al., 2014; Mackey et al., 2016) requiring increased cortical activity to overcome potential spinal inhibition. The increased knee flexor force-matching neural activity could also be secondary to the task being more atypical, as concentric precise force control of the hamstrings is not as common to be engaged during locomotion, where the quadriceps is primarily engaged in concentric positioning and the hamstrings act eccentrically to decelerate before heel strike. Thus, the nature of the concentric force matching task may result in increased activation for knee flexion that would not be the case with an eccentric force-matching task (Koohestani et al., 2020).

## Limitations

This investigation was limited to a single joint position and an isometric contraction at a low force level, primarily to minimize head motion for fMRI. Possibly, synergist muscle groups that contribute to hip flexion or extension may reduce the ability to isolate the quadriceps for knee extension or the hamstring for knee flexion (though at the low force level required in this study, accessory muscle activity is unlikely), so future work may consider recording electromyography measures to ensure muscle group contributions. The force level was selected to ensure a sufficient fluctuation range to test force-sense but also keep head motion minimal. We used a low absolute value of 5 N, as opposed to a low relative force such as 5% of a maximal voluntary contraction. Prior works have employed both a ~5 N absolute threshold (Newton et al., 2008) and similar relative thresholds (Shanahan et al., 2015). As our sample was homogeneous in terms of fitness, activity level, age, and BMI, there is a minimal indication the results would be different if scaled to a relative % for capability. Nonetheless, future work across varied samples may consider employing a relative metric for the force target. While we enrolled young and physically active females to better understand knee force control in this population at a unique high-risk for sensorimotor-related coordination errors that contribute to knee ligament injuries such as the anterior cruciate ligament, our participant selection criteria limit generalization to males or aging populations. Future investigations may consider heterogenous demographical recruitment of participants to increase generalizability or determine if changes in neural activity are present with various ages or pathological populations. Additionally, the use of a variety of joint angles and intensities may also highlight how limb position and magnitude plays a role in central mechanisms of force regulation.

## Conclusion

This investigation employed a novel lower extremity force-matching neuroimaging-compatible paradigm to examine motor control of the knee extensors and flexors. The paradigm was found to activate the sensorimotor network with unique neural correlates to force-matching error across parietal and frontal regions. This paradigm may allow for future research to better understand the neural correlates of lower extremity neuromuscular control across varied pathologies or interventions. Specifically, this foundational work can support a future investigation into the unique contribution of the nervous system to lower extremity force regulation in pathologies that disrupt proprioception and sensorimotor function such as knee anterior cruciate ligament injury (Laboute et al., 2019), osteoarthritis (Shanahan, 2015), and patella-femoral pain (Te et al., 2017). As the evidence base for the role of the nervous system in these musculoskeletal conditions grows, the need for such paradigms that bridge neural activity and motor performance of the knee as described here are needed to provide pathology specific therapeutic targets (Silfies et al., 2017; Armijo-Olivo, 2018).

## Data Availability Statement

The raw data supporting the conclusions of this article will be made available by the authors, without undue reservation.

## Ethics Statement

The studies involving human participants were reviewed and approved by Ohio University Institutional Review Board. The patients/participants provided their written informed consent to participate in this study.

## Author Contributions

DG, CC, and TW: conception. DG, CC, JS, and TW: experimental design and data analysis. DG, CC, AH, and TW: data collection. DG, CC, JS, AH, and TW: writing and review. All authors contributed to the article and approved the submitted version.

## Conflict of Interest

The authors declare that the research was conducted in the absence of any commercial or financial relationships that could be construed as a potential conflict of interest.
